# Epilepsy Phenotypes of Vitamin B6-Dependent Diseases: An Updated Systematic Review

**DOI:** 10.3390/children10030553

**Published:** 2023-03-15

**Authors:** Mario Mastrangelo, Valentina Gasparri, Katerina Bernardi, Silvia Foglietta, Georgia Ramantani, Francesco Pisani

**Affiliations:** 1 Child Neurology and Psychiatry Unit, Department of Human Neurosciences, Sapienza University of Rome, 00185 Rome, Italy; 2 Department of Neuroscience/Mental Health, Azienda Ospedaliero-Universitaria Policlinico Umberto I, 00161 Rome, Italy; 3 Department of Neuropediatrics, University Children’s Hospital Zurich and University of Zurich, 8032 Zurich, Switzerland

**Keywords:** pyridoxine-dependent epilepsies, metabolic epilepsies, PNPO deficiency, hyperprolinemia type II, PLPBP deficiency, GPI anchor defects, ALDH7A1 deficiency

## Abstract

Background: Vitamin B6-dependent epilepsies include treatable diseases responding to pyridoxine or pyridoxal-5Iphosphate (ALDH7A1 deficiency, PNPO deficiency, PLP binding protein deficiency, hyperprolinemia type II and hypophosphatasia and glycosylphosphatidylinositol anchor synthesis defects). Patients and methods: We conducted a systematic review of published pediatric cases with a confirmed molecular genetic diagnosis of vitamin B6-dependent epilepsy according to PRISMA guidelines. Data on demographic features, seizure semiology, EEG patterns, neuroimaging, treatment, and developmental outcomes were collected. Results: 497 published patients fulfilled the inclusion criteria. Seizure onset manifested at 59.8 ± 291.6 days (67.8% of cases in the first month of life). Clonic, tonic-clonic, and myoclonic seizures accounted for two-thirds of the cases, while epileptic spasms were observed in 7.6%. Burst-suppression/suppression-burst represented the most frequently reported specific EEG pattern (14.4%), mainly in PLPB, ALDH7A1, and PNPO deficiency. Pyridoxine was administered to 312 patients (18.5% intravenously, 76.9% orally, 4.6% not specified), and 180 also received antiseizure medications. Pyridoxine dosage ranged between 1 and 55 mg/kg/die. Complete seizure freedom was achieved in 160 patients, while a significant seizure reduction occurred in 38. PLP, lysine-restricted diet, and arginine supplementation were used in a small proportion of patients with variable efficacy. Global developmental delay was established in 30.5% of a few patients in whom neurocognitive tests were performed. Conclusions: Despite the wide variability, the most frequent hallmarks of the epilepsy phenotype in patients with vitamin B6-dependent seizures include generalized or focal motor seizure semiology and a burst suppression/suppression burst pattern in EEG.

## 1. Background 

Vitamin B6 is involved in several neuronal processes and networks via its active form, i.e., pyridoxal-5^I^-phosphate (PLP) [1]. PLP acts as a coenzyme in different biochemical cascades, including amino acids and neurotransmitter metabolism, folate, and 1-carbon metabolism, protein and polyamine synthesis, carbohydrate and lipid metabolism, mitochondrial functions, and erythropoiesis [2]. PLP depletion is the main pathophysiological mechanism for different inborn errors of metabolism, including ALDH7A1 deficiency, PNPO deficiency, PLP binding protein deficiency, hyperprolinemia type II, hypophosphatasia, and glycosylphosphatidylinositol (GPI) anchor synthesis defects (Table 1) [3]. The pathomechanisms behind PLP depletion include the accumulation of metabolites inactivating PLP (ALDH7A1 deficiency), the impairment of PLP synthesis and recycling (PNPO deficiency), defects in the intracellular transport of PLP (PLPBP deficiency), or impaired transport of PLP into the brain (hypophosphatasia and GPI anchor defects) [4].

These diseases share complex and overlapping presenting phenotypes, including epileptic seizures responding to pyridoxine (100 mg intravenously or 30 mg/kg/day orally) or PLP (50–85 mg/kg/day orally) [4]. In some cases, this therapeutic response can be incomplete or delayed for up to 3 weeks and does not include manifestations other than seizures (e.g., neurodevelopmental impairment and systemic symptoms) [4]. The severity of seizures and developmental impairment ranges from mild to severe, with a strong correlation between the earlier onset of symptoms and worse developmental outcomes [4].

The history of vitamin B6-dependent epilepsies covers about seven decades, from 1954 (the first patients reported by Hunt et al.) to the 2000s (the discovery of disease-causing genes for most of these disorders) [4].

This review aims to provide updated data on the epilepsy phenotypes of patients with a confirmed molecular genetic diagnosis of diseases affecting vitamin B6 metabolism.

## 2. Search Methods

We conducted a systematic review of published pediatric cases with a confirmed molecular genetic diagnosis of vitamin B6-dependent epilepsy according to PRISMA guidelines. A PubMed search was performed using the search terms “pyridoxine dependent epilepsy”, “ALDH7A1 deficiency”, “PNPO deficiency”, “PLPB binding protein deficiency” or “PROSC”, “hyperprolinemia type II”, “hypophosphatasia and “B6-responsive GPI anchor synthesis defects” and filtered results for the pediatric age and the temporal range 2000–2022. Reference lists of each selected article and systematic reviews with the same focus were reviewed to collect additional papers.

Articles reporting patients without a molecular genetic diagnosis, articles with no detailed info about seizure semiology, EEG patterns, response to therapy and/or clinical outcome, articles about studies not focusing on human beings, and articles that were not written in English were excluded from the analysis. 

Results were screened by title, abstract, and full text. Duplicates were excluded. Studies reporting overlapping cohorts were identified by comparing relevant features (i.e., number of subjects, time variables, outcomes, institution, and year). The studies with the most extensive reporting were selected for inclusion in our analysis. 

Data on demographic features, seizure semiology, EEG patterns, neuroimaging, treatment, and developmental outcomes were collected for each of the above-mentioned diseases.

**Figure 1:**
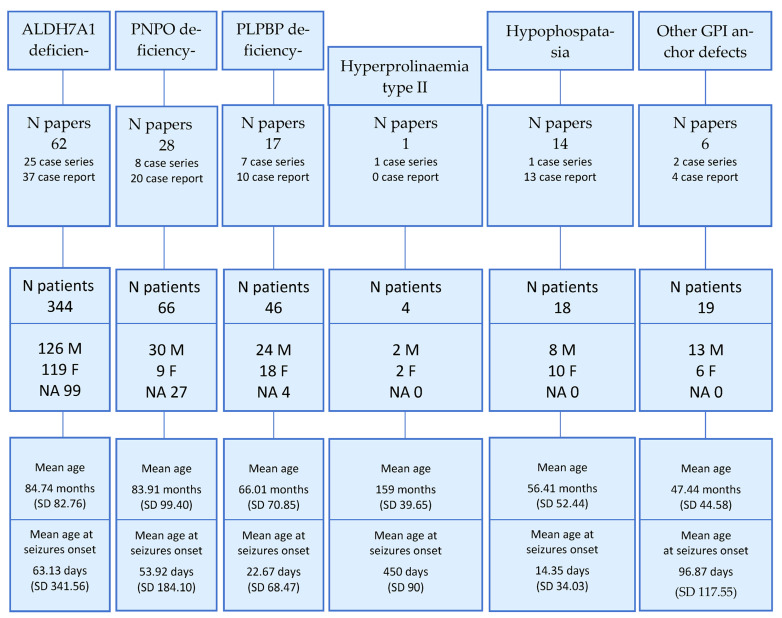
Flowchart reporting the selections of articles for this review.

Figure 1 summarizes the selected articles’ distribution and the reported patients’ main demographic data. Supplementary File S1 includes the data collection sheet.

The protocol of this systematic review is registered on the PROSPERO website (https://www.crd.york.ac.uk/PROSPERO/) (CRD42023396129 accessed on 1 February 2023).

## 3. Results

### 3.1. Patient Characteristics

Our search strategy yielded 497 patients with a confirmed genetic diagnosis of B6-dependent epilepsies. Of these, 90.7% (431/497) were diagnosed with the following three disorders: ALDH deficiency (69.4%), PNPO deficiency (13.2%), and PLPBP deficiency (9.2%) (Figure 1) [5,6,7,8,9,10,11,12,13,14,15,16,17,18,19,20,21,22,23,24,25,26,27,28,29,30,31,32,33,34,35,36,37,38,39,40,41,42,43,44,45,46,47,48,49,50,51,52,53,54,55,56,57,58,59,60,61,62,63,64,65,66,67,68,69,70,71,72,73,74,75,76,77,78,79,80,81,82,83,84,85,86,87,88,89,90,91,92,93,94,95]. Table 2 summarizes the differences among the patients with the various vitamin B6-dependent forms in terms of demographic and clinical features.

The mean age at inclusion in the respective study was 79.7 ± 81.8 months (data available for 307 patients) (Figure 1). A mild and not statistically significant prominence of males among the cases of PNPO deficiency, PLPBP deficiency, and GPI anchor defects was reported (Figure 1). A single late-onset case of hyperprolinemia type II, diagnosed at the age of 64 years, was excluded from the analysis [96]. Twenty-seven reported patients died during the reported follow-up period (Table 2, Supplementary File S1). 

Seizure semiology at onset was reported in all patients, while published detailed descriptions of seizure types during follow-up were available for only 18.7% of patients (Table 2, Supplementary File S1).

Global developmental delay was reported in 152 patients, while normal neurocognitive development was established in 94 cases (Table 2, Supplementary File S1). In other cases, the selective impairment of isolated functions (motor developmental delay and normal IQ in 4 patients with ALDH7A1 deficiency, isolated language delay in 11 patients) was demonstrated (Supplementary File S1). Neurocognitive tests were available for 50 patients only. IQ was below 70 in 19 patients, borderline (71–84) in 14, and higher than 85 in 17 (Table 2). Autism spectrum disorder was diagnosed in 4 patients (Table 2).

Initial EEG patterns were available for 197/497 patients, while longitudinal EEG data were collected for 143/497 patients (Table 2, Supplementary File S1). 

MRI was performed in 241/497 published patients. Potentially epileptogenic lesions, such as cortical dysplasia or other cortical malformations, mesial temporal sclerosis, hemorrhage, stroke, and hypoxic-ischemic encephalopathy, were detected in 39 patients; 111 had no neuroradiological abnormalities (Table 2, Supplementary File S1).

### 3.2. Epilepsy Phenotype

The mean age at seizure onset was 59.8 ± 291.6 days (data available for 429/497 patients), with earlier onset in hypophosphatasia and PLPBP deficiency (Table 2; Supplementary File S1).

Seizure onset mainly occurred in the neonatal period (67.8% of cases), while only ten patients presented with seizures after the age of 12 months (four with ALDH7A1 deficiency, three with PNPO deficiency, one with PLPBL, and two with hyperprolinemia type II) (Table 2, Supplementary File S1). 

Generalized motor seizures were the most frequently reported initial seizure type (148 cases). In contrast, focal motor seizures occurred at epilepsy onset in 108 patients. Complex presentations, including both focal and generalized manifestations, were described in 26 (Table 2, Supplementary File S1). Clonic, tonic-clonic, and myoclonic seizures accounted for about two-thirds of cases, while epileptic spasms were observed in 38 cases (20 with ALDH7A1 deficiency) (Table 2, Supplementary File S1). Thirty-eight patients had a history of febrile seizures, while epilepsy manifested with status epilepticus in 36 (Table 2, Supplementary File S1). 

Four patients carrying pathogenic variants of ALDH7A1 and one with PNPO deficiency developed no seizures because of an early pre-symptomatic supplementation since birth, following prenatal diagnosis after previous familial cases (Table 2, Supplementary File S1).

No specific seizure type had a significant predominance during follow-up. However, only a few reports included adequate descriptions of age at seizure onset, seizure frequency, and responsiveness to antiseizure medication (Table 2, Supplementary File S1). 

Burst-suppression/suppression-burst represented the most frequently reported specific EEG pattern (72 cases), mainly in PLPB, ALDH7A1, and PNPO deficiency (Table 2, Supplementary File S1). 

Hypsarrhythmia was observed in 13 patients (7 with PNPO deficiency) (Table 2, Supplementary File S1). Paroxysmal interictal abnormalities were reported in 65 patients, while non-paroxysmal abnormalities (including EEG background disorganized for age, background slowing, and other nonspecific alterations) were recorded in 45 patients (Table 2, Supplementary File S1). No EEG interictal abnormalities were reported in 18 patients (Table 2, Supplementary File S1).

No significant EEG abnormalities were observed in 63 patients after the beginning of vitamin B6 supplementation (Supplementary File S1). Burst suppression and hypsarrhythmia persisted after the initial treatment in seven and six patients, respectively (Supplementary File S1). Paroxysmal abnormalities were reported in 65 cases (Supplementary File S1).

### 3.3. Treatment

Pyridoxine was administered to 312 patients (58 intravenously, 240 orally, and 14 not specified) (Table 2, Supplementary File S1). Dosages ranged between 1 and 55 mg/kg/die (Supplementary File S1). Complete seizure freedom was observed in 173 patients, while a significant seizure reduction occurred in 38 patients (Table 2, Supplementary File S1). Three patients received an intrauterine pyridoxine supplementation according to a pre-symptomatic therapeutic strategy after a prenatal diagnosis in affected families (Supplementary File S1). 

Twenty patients received a lysine-restricted diet, while arginine supplementation was reported in eleven (Table 2). Twelve patients received triple therapy, including a combination of pyridoxine, a lysine-restricted diet, and arginine supplementation (Table 2).

PLP was administered in 45 patients (mainly with PNPO or PLPBP deficiency), with dosages ranging between 10 and 85 mg/kg/die (Supplementary File S1). The administration of PLP resulted in complete seizure control in only five patients (Table 2, Supplementary File S1).

Various antiseizure medications were administered with the goal of attaining seizure control in 213 patients (Supplementary File S1). However, the proportion of patients in whom subsequent B6 supplementation allowed to withdraw other pharmacological therapies cannot be easily inferred from these past studies.

## 4. Discussion

B6-dependent epilepsies may present with different seizure types, including focal and bilateral clonic, myoclonic, tonic, and atonic seizures, and epileptic spasms, with semiology gradually changing, variable seizure types occurring and becoming more prominent over time [2,4,97]. The same degree of variability was observed in EEG patterns, which mainly included continuous or intermittent focal, multifocal, or generalized epileptiform discharges with (rhythmic) sharp waves of unilateral or bilateral distribution, a burst suppression pattern, and a bilateral slow delta activity [98]. It should be noted that no apparent interictal or ictal abnormalities were identified in the initial EEG in some cases of B6-dependent epilepsies [98]. 

Age at seizure onset did not differ significantly between ALDH7A deficiency and other B6-dependent epilepsies [1,16]. Seizure onset after one month accounted for up to one-third of patients [7,14,97]. Less severe developmental impairment was reported in patients with seizure onset between 3 and 12 months [7,14,97].

This variability has prompted experts to recommend therapeutic trials with pyridoxine or PLP in all neonatal seizures, including seizures of unknown etiology with a partial, transient, or lacking response to antiseizure medication [1]. Our systematic analysis of patients with confirmed molecular genetic diagnosis of vitamin B6-dependent disorders provides a reliable panorama of epilepsy phenotypes, where an early therapeutic trial may result in a better outcome for treated patients. The most often reported candidate profiles for B6 supplementation include patients under one month with generalized or focal motor seizures and newborns with burst-suppression/suppression-burst patterns [4]. The occurrence of sequential seizures in the neonatal period may represent a significant part of the phenotype in many patients (Supplementary File S1). Although different seizure types, such as focal and bilateral clonic, myoclonic, tonic and atonic seizures, and epileptic spasms, have been reported within the context of B6-dependent epilepsies, there is a dearth of data regarding their timing of occurrence and order of precedence during the course of the disease (Supplementary File S1, Table 2). Infantile spasms associated with hypsarrhythmia accounted for a lower number of initial electroclinical patterns in all vitamin B6 diseases, suggesting that supplementation with pyridoxine or PLP should be considered as second-line treatment, after ACTH and vigabatrin, in these cases [92,99]. Epileptic spasms were also recently reported without an associated hypsarrhythmia in a 5-month-old infant presenting with combined paroxysmal eye-movement disorders and carrying a previously unreported bi-allelic pathogenic PLPBP variant [100].

Atypical Dravet-like clinical patterns were reported in two patients, but no further details about the clinical course or the response to treatment were provided [91]. A significant proportion of patients reported febrile seizures or recurrent episodes of status epilepticus during acute febrile infections, especially in those with ALDH7A1 deficiency. These events were generally not prevented by the transient increase of pyridoxine dosage during maintenance therapy [91,101]. Absences and atonic seizures, not considered typical of B6-dependent seizures, were reported in a few patients [25,41]. Status epilepticus accounted for about 8% of initial clinical presentations in patients with known genetic etiology (Table 1). Focal clonic status epilepticus was considered unusual in the past but appeared to have the same frequency as other forms [7,102].

Supplementation with pyridoxine was preferred to PLP in most cases, independently from the molecular genetic defect, and resulted in a higher rate of seizure-free patients. Pyridoxine is usually preferred because it is more easily commercially available in almost all countries, but also because different clinical observations highlighted its remarkable efficacy in diseases previously considered responsive exclusively to PLP (e.g., PNPO deficiency) [103,104]. These advantages were confirmed in cases of PNPO deficiency, where the change from pyridoxine to PLP resulted in seizure aggravation or even the occurrence of status epilepticus in affected children [81,103,104]. Lysine-restricted diet and arginine supplementation offered interesting perspectives about the possibility of modifying neurocognitive development. However, the follow-up duration of cohorts receiving these treatments is too short to evaluate their efficacy in achieving this aim (Table 2, Supplementary File S1) [83].

**Figure 2:**
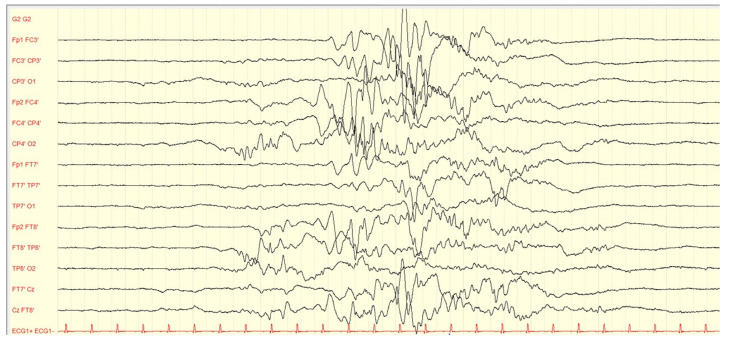
Burst suppression EEG trace of a neonate with PNPO deficiency who was born at 37 weeks gestational age and presented with status epilepticus on the first day of life. The neonate was treated first with 40 mg/kg levetiracetam and then with 5 mg/kg phenobarbital with only partial response. Next, according to our institutional protocol, we administered pyridoxine 100 mg i.v., followed by a maintenance dosage of 30 mg/kg/day. The introduction of pyridoxin led to seizure freedom and EEG improvement. Highpass: 0.530 Hz, Lowpass: 70 Hz.

**Figure 3:**
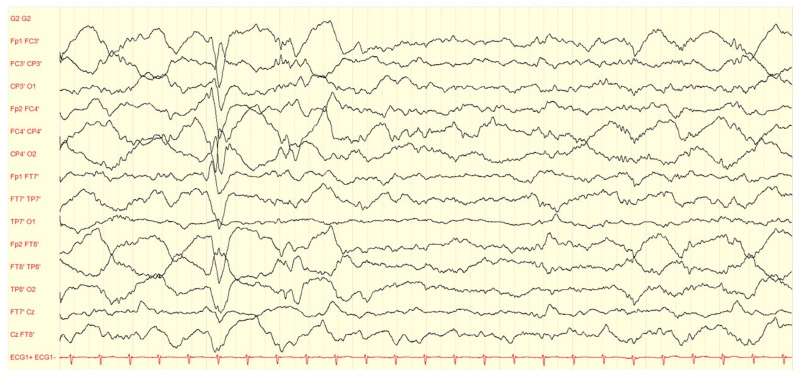
Continuous EEG trace of the same neonate of Figure 2 at age one week. Seizure freedom and EEG improvement after initiating pyridoxine treatment correlated with neurological improvement (less lethargic, needs less O2, better feeding). Highpass: 0.530 Hz, Lowpass: 70 Hz.

**Figure 4:**
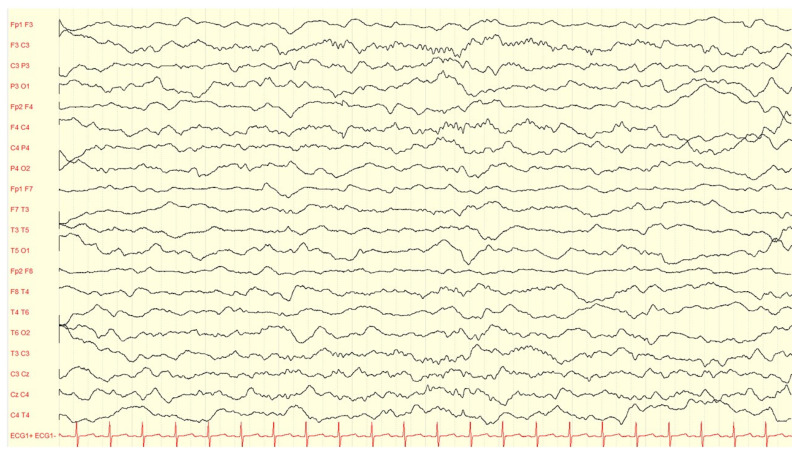
Symmetric spindles in sleep EEG in the same neonate of Figure 2 and Figure 3 at age two months under continued levetiracetam 15 mg/kg/day and pyridoxal phosphate 15 mg/kg/day.

EEG has been historically considered a valuable tool in evaluating the effect of pyridoxine or PLP administration in children with B6-dependent epilepsies. Figure 2, Figure 3 and Figure 4 present illustrative EEG recordings over the course of the disease in a case of PNPO deficiency diagnosed in our institution, which presented on the first day of life with refractory status epilepticus, burst suppression in EEG, lethargy, need for O2 supplementation, and poor feeding, to be followed by a gradual recovery, including seizure control, EEG normalization, and neurological improvement, after B6 supplementation. However, several authors reported that burst suppression patterns and other trace abnormalities often persist after clinical seizures have been stopped and, in several cases, after weeks or months of pyridoxine maintenance therapy [98]. The EEG response to the intravenous administration of pyridoxine is no longer considered a criterion to confirm or exclude the diagnosis of the different genetically determined B6-dependent epilepsies [39]. In some cases, interictal EEG traces may be expected before and/or after the initiation of treatment with pyridoxine [16,91].

The impact of available treatment is apparently more significant in epilepsy than in neurocognitive development, although the extreme heterogeneity of outcome measures, ranging from mere parental reports to formal cognitive testing, does not allow drawing any reliable or generalizable conclusions on these aspects [76]. Interestingly, the early initiation of pyridoxine and lysine reduction therapies resulted in a significant increase of 21.9 points in the developmental quotient in a recent study including 60 cases of B6-dependent epilepsies [105], thus opening new and encouraging perspectives in this field. It should, however, be noted that the observed improvement in cognitive development did not differ significantly between children receiving both pyridoxine and lysine and those receiving only pyridoxine in this study [105]. Another retrospective multicenter cohort study considering 18 families with B6-dependent epilepsies showed that children who had an early initiation of pyridoxine supplementation achieved better performances in fine motor skills compared with their siblings who had a later initiation of pyridoxine supplementation [74]. The early introduction of combined pyridoxine and lysine reduction therapies resulted in optimized profiles in neurodevelopmental, cognitive, behavioral, and psychiatric domains in affected children [74]. 

The evolution of clinical, neurodevelopmental, and epilepsy phenotypes in adulthood has hardly been investigated so far. In a recent Dutch study investigating a young adult cohort, including 10 patients with ALDH7A1 deficiency at the age of 18 to 30 years (7 of 10 carrying a homozygous c.1279 G>C variant), good seizure control was reported [106]. Seizure breakthroughs in adult life are relatively infrequent and usually correlate with sub-optimal adherence to pyridoxine supplementation [106]. In the abovementioned cohort, a significant variability of cognitive functions was observed, with one patient notably attending university [106]. 

The limitations of the present systematic review include possible bias due to the retrospective nature of the collected data and the exclusion of patients with pyridoxine dependency without a confirmed molecular genetic diagnosis. This last factor may also represent a point of strength because it allowed for the analysis of the phenotypes that were undoubtedly associated with specific genetic defects.

## 5. Concluding Remarks

Despite the enormous variability, this systematic review depicted the features of the epilepsy phenotype in patients with vitamin B6-dependent seizures. The systematic review confirmed that generalized or focal motor seizures and burst suppression/suppression burst patterns are the most frequent hallmarks of these conditions. A worldwide patient registry is ongoing, and 130 patients have been reportedly recruited between 2014 and 2021 (www.pdeonline.org) (accessed on 1 March 2022). The international consortium that manages the registry aims to expand the knowledge concerning the clinical features and the genetic basis of these disorders and, most importantly, to promote the optimization of actual and future therapeutic protocols in B6-dependent epilepsies.

Indeed, the most relevant open questions for the few next years include several aspects of clinical phenotypes and therapeutic options, including: (a) Better characterization of late-onset or atypical phenotypes (seizure onset after the age of 3 months, milder epilepsy phenotypes, less severe developmental impairment); (b) better definition of effective therapeutic pyridoxine dosages, especially for oral administration, to be increasingly used also in the acute phase; (c) more detailed studies about the pre- and postnatal prophylactic treatments of siblings of children with previously confirmed pyridoxine-dependent epilepsy; (d) definition of evidence-based therapeutic protocols, including lysine-restricted diet and arginine supplementation administered from the early stage onward; and (e) therapeutic strategies addressing the prevention of progressive developmental impairment.

## Figures and Tables

**Table 1:** Epidemiological, clinical, biochemical, genetic, and neuroimaging features of vitamin B6-dependent diseases.

DISEASE (OMIM)	ESTIMATED PREVALENCE	GENE	CLINICAL FEATURES	BIOCHEMICAL MARKERS	NEUROIMAGING FEATURES	THERAPEUTIC OPTIONS
ALDH7A1 DEFICIENCY	1:20,000–600,000	ALDH7A1 MIM #266100	Neonatal seizure (focal and/or generalized, status epilepticus) only few cases of later onset Other features: Abnormal foetal movements, signs of hypoxic ischaemic encephalopathy, dystonia, increased startle response, irritability, intellectual disability, respiratory distress, abdominal distension, bilious vomiting, hepatomegaly, hypothermia, shock, and acidosis	Urinary α-AASA, urinary or plasma α- AASA/P6C ratio;	Decreased mielinization, periventricular leukomalacia, atrophy, dysplasia, and other white matter abnormalities, gliosis, arachnoid and subependimal cysts, mega cisterna magna, hydrocephalus, ventriculomegaly, wide extracerebral CSF spaces, cerebral atrophy, grey matter heterotropia, cortical displasies, mesial temporal sclerosis, cortical malformation, cerebellar hypoplasia/ atrophy, cerebellar wide posterior fossa, chiari I, hemorragies, hematoma, hipoxic-ischemic like encephalophaty signs, thinning/hypoplasia/ dysplasia/ agenesia of the corpus callosum	Acute: PN (iv): 50–300 mg Maintenance:PN (os): 25–900 mg/day (1.3−70 mg/kg/day) Lysine restricted diet Arginine supplementation
PNPO DEFICIENCY	1–9/1,000,000	PNPO MIM #610090	Neonatal seizures (focal and/or generalized, status epilepticus) only few cases of later onset Other features: Dystonia, prematurity-related disorders, signs of metabolic derangement (including metabolic acidosis, hyperlactacidaemia, hypoglycaemia), anaemia, microcephaly and gastrointestinal symptoms (abdominal distension and hepatomegaly, autism		Ventriculomegalia, white matter abnormalities, decreased mielinization, periventricular leukomalacia, gliosis, cerebral atrophy, cortical malformation, hipoxic-ischemic like encephalophaty	Acute: PLP: 50–200 mg; PN: 50–600 mg Maintenance: PLP: 10–72 mg/kg/day; PN: 5.5–50 mg/kg/day
PLPBP DEFICIENCY	unknown	PLPBP MIM #617290	Neonatal seizure (focal and/or generalized, status epilepticus) only few cases of later onset Other features: Movement disorders, microcephaly, global developmental delay, autism		White matter abnormalities, decreased mielinization, cystic leukencephalopaty, ventriculomegalia, cerebral atrophy, mesial temporal sclerosis, cortical malformation, thinning of the corpus callosum	Acute: PN: 50–200 mg Maintenance: PLP: 10–58 mg/kg/day PN: 4.7–24 mg/kg/day
HYPERPROLINAEMIA TYPE II	unknown	ALDH4A1 MIM #239500	Seizure onset after neonatal period (generalized) other features: behavioral disturbances, intellectual disability; Possible asymptomatic forms	Plasma proline, urinary Δ-1-pyrroline- 5-carboxylate, Δ-1-pyrroline- 5-carboxylate dehydrogenase activity in leukocytes and skin fibroblast;	/	Acute: PN (iv): 110 mg Maintenance: PN (os): 50–150 mg
HYPOPHOSPHATASIA	unknown	ALPL MIM #241500	Neonatal seizure (generalized) Other features: Developmental delay, Dysmorphisms, Sheletal abnormalities including brachy-telephalangy		cerebral atrophy, ventriculomegalia, white matter abnormalities, white matter atrophy	Acute: PN (iv): 25–200 mg Maintenance: PN: 10–30 mg/kg/day (25–160 mg/day)
GPI ANCHOR SYNTHESIS DEFECTS	unknown	PIGO NGS) MIM 614730 PIGV MIM # 610274 PIGA MIM # 311770 PIGQ MIM # 605754 PIGC MIM # 601730 PIGH MIM # 600154 PIGP MIM # 605938 PIGY MIM # 610662 PIG E MIM 610274	Seizure onset after neonatal period (focal and/or generalized) other features: developmental delay intellectual disability		decreased mielinization, white matter abnormalities, wide extracerebral CSF spaces, cerebral atrophy, cerebellar atrophy, thinning and hipoplasia of the corpus callosum	Acute:PN (IV): 100 mg Maintenance: PN: 100–400 mg (20–30 mg/kg/day)

**Table 2:** Demographic, developmental, and epileptological features of published patients with vitamin-dependent diseases.

	ALDH7A1 DEFICIENCY	PNPO DEFICIENCY	PLPBP DEFICIENCY	HYPERPROLINAEMIA TYPE II	HYPOPHOSPHATASIA	GPI ANCHOR SYNTHESIS DEFECTS
Number of patients (M and F)	344 (126 and 119)	66 (30 and 9)	46 (12 and 10)	4 (2 and 2)	18 (8 and 10)	19 (13 and 6)
Death during the follow-up	5	5	7	-	6	4
Mean age at the onset of seizures (days)	63.13	53.92	22.67	450	14.35	96.87
Patients with onset before the age of 1 month	222	55	39	-	16	5
Patients with onset after the age of 1 month	62	10	5	3	1	11
Patients with generalized motor seizures at onset	92	20	20	1	10	5
Patients with focal motor seizures at onset	77	12	11	-	1	7
Patients with epileptic spasms at the onset	20	11	6	-	-	1
Patients with febrile seizures at the onset	27	5	2	1	1	2
Patients with status epilepticus at the onset	25	8	2	1	-	0
Patients with generalized motor seizures during the follow-up	15	8	4	1	4	8
Patients with focal motor seizures during the follow-up	10	7	1	-	1	3
Patients with generalized non-motor seizures during the follow-up	-	-	-	1	1	0
Patients with focal non-motor seizures during the follow-up	-	-	-	-	-	1
Patients with epileptic spasms during the follow-up	3	3	-	-	-	7
Patients with febrile seizures during the follow-up	11	2	7	-	1	0
Patients with status epilepticus during the follow-up	13	5	2	-	1	5
Patients with global developmental delay	103	19	19	1	1	9
Patients with selective language disorder	6	3	3		-	0
Patients with IQ higher than 85	64	19	10	1	-	0
Patients with borderline IQ (71–84)	13	-	1	3	-	0
Patients with IQ below 70	35	9	5	0	-	0
Patients with autism spectrum disorders		2	2	0	-	-
Patients with suppression burst pattern at EEG	21	18	27	-	4	2
Patients with suppression hypsarrhythmia at EEG	3	7	2	-	-	1
Patients with paroxysmal abnormalities at EEG	25	14	14	-	3	9
Patients with non-paroxysmal abnormalities at EEG	11	9	19	-	2	4
Patients with no EEG abnormalities at the initial EEG	14	1	1	-	2	0
Patients with no EEG abnormalities during the follow up	41	15	6	0	1	0
Patients with MRI available	151	30	34	0	9	17
Patients with epileptogenic lesions at MRI	27	4	8	0	0	0
Patients responsive to pyridoxine	122	23	20	2	4	2
Patients responsive to PLP	1	2	2	-	-	-
Patients treated with lysine restricted diet	20	-	-	-	-	-
Patients treated with triple therapy	12	-	-	-	-	-
Patients treated with other antiseizure medications	117	31	33	3	15	14

## Data Availability

Collected data are summarized in File S1.

## Supplementary Materials

The following supporting information can be downloaded at: https://www.mdpi.com/article/10.3390/children10030553/s1, File S1: Datasheet summarizing all the literature data collected for this review.
